# A Lightweight Hierarchical Activity Recognition Framework Using Smartphone Sensors

**DOI:** 10.3390/s140916181

**Published:** 2014-09-02

**Authors:** Manhyung Han, Jae Hun Bang, Chris Nugent, Sally McClean, Sungyoung Lee

**Affiliations:** 1 Ubiquitous Computing Laboratory, Department of Computer Engineering, Kyung Hee University, 1 Seocheon-Dong, Giheung-Gu, Yongin-Si, Gyeonggi-Do 446-701, Korea; E-Mails: smiley@oslab.khu.ac.kr (M.H.); jhb@oslab.khu.ac.kr (J.H.B.); 2 School of Computing and Mathematics, Computer Science Research Institute, University of Ulster, Newtownabbey, Co. Antrim, BT37 0QB, UK; E-Mail: cd.nugent@ulster.ac.uk; 3 School of Computing and Information Engineering, University of Ulster, Coleraine, Co. Londonderry, BT52 1SA, UK; E-Mail: si.mcclean@ulster.ac.uk

**Keywords:** activity recognition, smartphone, multimodal sensors, naïve Bayes, life-log

## Abstract

Activity recognition for the purposes of recognizing a user's intentions using multimodal sensors is becoming a widely researched topic largely based on the prevalence of the smartphone. Previous studies have reported the difficulty in recognizing life-logs by only using a smartphone due to the challenges with activity modeling and real-time recognition. In addition, recognizing life-logs is difficult due to the absence of an established framework which enables the use of different sources of sensor data. In this paper, we propose a smartphone-based Hierarchical Activity Recognition Framework which extends the Naïve Bayes approach for the processing of activity modeling and real-time activity recognition. The proposed algorithm demonstrates higher accuracy than the Naïve Bayes approach and also enables the recognition of a user's activities within a mobile environment. The proposed algorithm has the ability to classify fifteen activities with an average classification accuracy of 92.96%.

## 1. Introduction

Activity Recognition (AR) solutions are capable of identifying physical actions, postures or movements using various sensors which have the ability to capture the intention and the condition of the current situation of the subject. Existing AR research has utilized wearable sensors which have been attached on the human's body or 2D/3D cameras for the purposes of capturing video-based images [1,2]. A range of studies have also considered the use of mobile phones for the purposes of AR [3–5]. In those studies, the data is collected using a mobile phone and the AR is normally performed offline. Thus, the AR algorithms are not implemented in real-time on the phone and the classification is not performed in real-time. In [6], an AR system running purely on a smartphone is presented. The system can be trained on the mobile phone itself and also has the ability to perform the data classification in real-time on the phone. The recognition is based on features calculated using a geometric template matching approach and a support vector machine (SVM) is used as the classifier.

There are several advantages of smartphone-based AR. The first one is the availability of various sensor devices already embedded in the phones, so the users are not required to use external sensors in order to collect activity information. The flexibility and readiness of the smartphone as a sensor device are also seen as advantages. The second one is that the smartphones already have many properties that enable AR related implementations. They provide processing capability, adequate storage space and communication ability, *etc.* The third one is that a smartphone is likely to be with a user during daily activities, so the system easily acquires activity and context data from users in an unobtrusive way. The fourth and final advantage is that most smartphones also have relatively long operation duration. In order to recognize a whole day of a user's activities, a smartphone should have at least a day's worth of operation time before recharging is required. Current smartphones already have sufficient operating system properties and battery power reliability.

Smartphones have multiple embedded sensor devices and are able to process collected data independently. For recognizing physical movements of the user, accelerometer and gyroscope sensors can be utilized. For gathering contextual data such as location, situation or environmental information, data from GPS, proximity sensors and the sound microphone are also used. A smartphone also has enough storage space, processing capability and communication modules, hence is a good choice as a sensing and processing platform for recognizing a user's activities.

General AR is divided into two phases. Firstly a training phase is required to build the activity models and secondly a recognition phase where based on activity models the data collected is processed. Probability-based AR algorithms for example hidden Markov models (HMM), SVM or k-nearest neighbor (kNN) are difficult to apply to smartphone applications given that they requires sample data for modeling in addition to their computational complexity [7].

For a lightweight activity classification framework, the Naïve Bayes algorithm is used as a basic algorithm for recognizing human activities. If the activity information of users is matched to the candidate who has the highest possibilities among pre-constructed activity models, that one is chosen by the algorithm. The algorithm must be lightweight and fast, especially if the target platform is a mobile device such as a smartphone. A comparison of several classification methodologies is given in [8] and shows that the Naïve Bayes classifier achieves the fastest modeling time than other machine learning algorithms. With regard to comparing model generation time, Naïve Bayes shows the fastest modeling results among popular machine learning algorithms such as Decision Table (DT), Decision Tree (ID3), Tree-augmented Naïve Bayes (TAN), Artificial Neural Network (ANN). That is mainly due to the absence of ‘searching’ in the Naïve Bayes algorithm. Based on these findings, we have used a Naïve Bayes algorithm in real-time activity modeling and recognition on a smartphone in our current work.

Although the Naïve Bayes classifier generates an activity model quickly, it has several limitations such as relatively low processing speed, and difficulties to apply it in mobile environments which have less resources. First, in the modeling phase extracting features from collected data can result in many errors occurring due to insufficient memory allocation in the mobile device. Second, one of the inherent characteristics of the Naïve Bayes classifier is that every attribute has the same priority. This causes lower accuracy of the posterior probability, therefore in order to resolve the above problems, the Adaptive Naïve Bayes (A-NB) algorithm is proposed in the current work.

**Figure 1.**
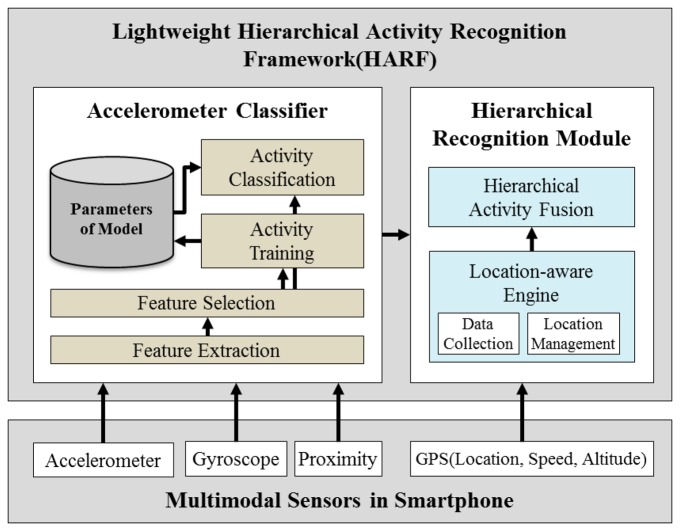
Overall architecture of the proposed system–HARF.

In this paper, a lightweight activity modeling and recognition framework defined as the Lightweight Hierarchical Activity Recognition Framework (HARF) which enables the modeling and recognition of user activities on a smartphone is proposed. The proposed HARF has the ability to recognize 15 activities and uses the accelerometer, gyroscope, proximity sensor and GPS modules that all come with the smartphone (Figure 1).

## 2. Related Works

**Figure 2.**
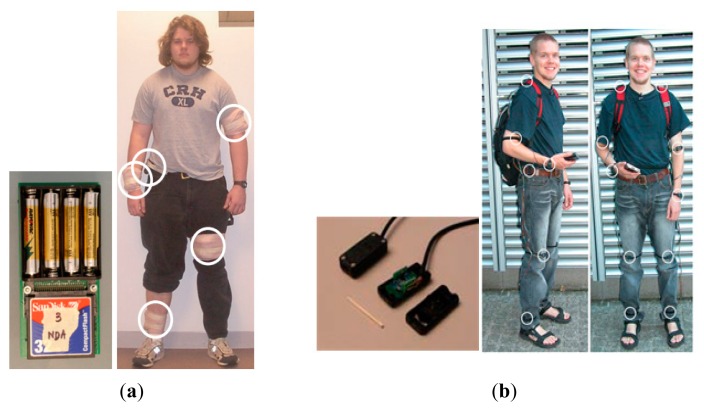
Sensor replacements in two activity recognition research studies using body-worn sensors. Figures are adapted from the corresponding papers [1,9].

Many of the popular AR approaches have proposed the use of body-worn sensors with different sensors commonly placed on different parts of the body. The following two examples have been selected to show how the placement of sensors and corresponding devices was proposed. Researchers in [1] used five sensor boards on different parts of the body such as arm, wrist, knee, ankle and waist. Each sensor board consisted of a biaxial accelerometer, four AAA batteries and a memory card for storage. As shown in Figure 2, the authors in [9] investigated activity recognition using 12 body-worn tri-axial accelerometers. Both investigations have shown that accelerometer-based AR can provide over 90% accuracy. Nevertheless, in order to enable the recognition of basic activities, the approaches suggest the use of additional sensors placed at fixed strategic positions depending on the targeted activities.

In [10], authors claim that such approaches are obtrusive for a person. In the investigation of [1], the authors mentioned that in some of the experiments participants have reported that they felt self-conscious in public spaces. This was due to the sensor devices used being visually noticeable. Researchers in [11] stated that the placement of sensors in multiple predefined locations could be quite obtrusive. They contended this as a limitation for common AR approaches using body-worn sensors.

Others have made similar observations on the same issue. In [12] the authors suggested the use of a single sensor placement as a less obtrusive alternative. The authors in [13] discussed this issue saying that wearable multi-sensor solutions are very obtrusive since wired techniques are used and were required most of the time and users have to strap sensors on with Velcro strips or even wear special suits for the intended AR. Instead of placing different sensors on the person's body for continuous monitoring, we propose to use unobtrusive and a minimum number of devices. The person should not consciously feel intruded or disturbed by the number or the placement of the sensors.

A smartphone can be seen as a potential unobtrusive sensor device. Currently, most smartphones onthe market have multiple built-in sensors, such as accelerometers, microphones, proximity sensors, GPS and light sensors. Particularly in the case where the person already owns a smartphone, it isnot necessary for them to use an additional device for sensor data collection. If there is such ademand in the near future, additional external sensor devices can still be connected wirelessly tothe smartphone for data collection, processing, transfer and even evaluation. Smartphones can beused as alternatives to current body-worn sensor devices based on the following factors:

➀ *The available sensors are built-in*. As long as the desired context can be derived and recognized from the data of the built-in sensors, the users are not required to use any external sensors in order to collect the required information. In cases where the need occurs, additional sensors and devices can be interfaced to smartphones to extend their functions with the necessary sensors other than those which are built-in. The flexibility and readiness of the smartphone as a sensor device are viewed as advantages.
➁ *Smartphones already have many properties that enable activity recognition-related implementations*. Most smartphones have relatively high processing power and sufficient memory for data processing tasks. They also contain more than adequate storage space for the storage of raw and computed data. Smartphones also provide communication possibilities that allow information exchange between users and external services. The smartphone itself can be viewed as a small computing device with common connectivity integrated.
➂ *A smartphone is likely to be with a user during daily activities*. It can be considered as a natural choice of an unobtrusive device. The chances of users feeling awkward or uncomfortable will be much lower compared to approaches that affect the usage habits of the users.
➃ *Most smartphones have also relatively long operation durations*. For an average user, under normal usage patterns (some daily phone conversations and text messages), a smartphone should have at least a day's operation time before a recharge is required. With proper management for sensor data polling, an entire day of sensor data collection and processing may therefore be achievable.

There are several existing studies for activity modeling and recognition algorithms. In [14], the authors used multiple sensors or heterogeneous sensors for recognizing user's activities by attaching them to the body. The approach required a physical connection between sensor devices; however, such an approach is not suitable for long-term AR. For accelerometer data classification, several approaches to feature extraction and classification have been investigated [15]. Nevertheless, existing approaches are difficult to apply to mobile devices which have relatively less resources than computers or servers for the initial stages of training.

## 3. The Proposed Algorithm and Systems

In this paper the Naïve Bayes algorithm is used as a basic algorithm for recognizing a human's activities. If the activity information of users is matched to the model which has the highest possibilities among pre-constructed activities models, it is chosen by the algorithm. Generally the Naïve Bayes classifier achieves faster modeling time and less computation overheads than other machine learning algorithms. The Naïve Bayes classifier can generate an activity model quickly, however, it has several limitations such as relatively low processing speed and it is difficult to deploy in a mobile environment which is resource constrained. In addition, one of the inherent characteristics of the Naïve Bayes classifier is that every attribute has the same priority, which results in a lower accuracy of the posterior probability. In the current work, an Adaptive Naïve Bayes approach is proposed in an effort to address the aforementioned issues and a lightweight hierarchical activity recognition framework is proposed based on an Adaptive Naïve Bayes approach.

### 3.1. Adaptive Naïve Bayes Algorithm (A-NB)

Naïve Bayes is a statistical classification method which can estimate the possibility of a given sample. The Naïve Bayes probabilistic model assumes that sample data *F*_1_ to *F_n_* have possibilities relating to an independent class *C*. The probability of *C* after the sample data *F*_1_… *F_n_* are collected is *p*(*C*|*F*_1_,…, *F_n_*) which is referred to as the posteriori probability. In order to calculate *p*(*C*|*F*_1_,…, *F_n_*), *p*(*F*_1_,…, *F_n_*) and *p*(*C*) are required. These can be estimated from training data and are referred to as the boundary probability. By using Bayes's theorem the *a posteriori* probability is defined by Equation (1):

(1) $$p(C|F_{1},\ldots ,F_{n})=\frac{p(C)p(F_{1},\ldots ,F_{n}|C)}{p(F_{1},\ldots ,F_{n})}$$

Consider a maximization of*p*(*C*)*p*(*C*|*F*_1_,…,*F_n_*) because *p*(*F*_1_,…,*F_n_*) has values to every class. If the boundary probability of the class is now known, only *p*(*F*_1_,…, *F_n_*|*C*) may be considered. *p*(*F*_1_,…, *F_n_*|*C*) is calculated by the independent assumption of Naïve Bayes. As a result, *F*_1_,…, *F_n_* can be classified as the class which has the largest *a posteriori* probability. If a sample data *F_i_* is a classification attribute and contains one value out of several limited values, a calculation of *p*(*F_i_*|*C*) may be made according to traditional probability. Nevertheless, the characteristic of the training data for AR is continuous data. In this case, the distribution of probability is utilized for calculating the conditional probability and the Gaussian distribution is utilized for representing a distribution of *F_i_*:

(2) $$P(F_{i}=v|C)=\frac{1}{\sqrt{2\pi \sigma_{C}^{2}}}e^{-\frac{{\left(v-\mu_{C}\right)}^{2}}{2\sigma_{C}^{2}}}$$

In Equation (2), the mean value of *F_i_* in class *C* is *μ_c_* and the distribution is $\sigma_{c}^{2}$.

**Figure 3.**
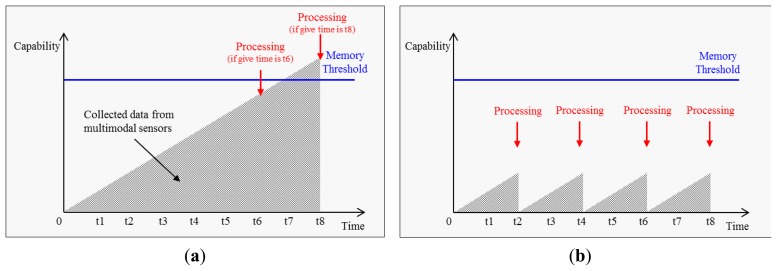
(**a**) Memory usage using Naïve Bayes; (**b**) Memory usage and processing frequency using adaptive Naïve Bayes.

In the current work, the proposed approach utilizes multiple sensor data gleaned from a smartphone. Taking this into consideration a lightweight modeling and recognition algorithm is therefore required due to the limited resources. Mobile devices, including smartphones, have a limited memory space for storing and processing collecting raw sensor data in real-time. This causes memory overflow problems as described in Figure 3a. To avoid the memory overflow problem, the adaptive approach, repetitive calculation, which is periodically calculate accumulated sensor data is proposed as depicted in Figure 3b. A-NB keeps calculations to every certain period time *t*. This lowers the allocated memory and increases the processing frequency. After collection of raw sensor data is completed, all of the temporarily calculated results are utilized for activity modeling.

In addition, given the performance issues associated with the Naïve Bayes approach a lightweight classification algorithm A-NB, which enables activity modeling and recognition in a smartphone, is proposed. When a system builds an activity model using Naïve Bayes, the complexity of the calculation is dependent on the number of sample data *i*.

If the AR features are increased, this requires significant processing capabilities whilecalculating the mean value *μ_c_* and the distribution $\sigma_{c}^{2}$ of data *F_i_*. To overcome memoryoverflow problems which may occur during real-time activity training, A-NB calculates the mean and distribution values of data *F_i_* periodically. The repetitive approach considering memory usage and efficiency is described below:

(3) $$\begin{array}{c} \mu_{N}=\frac{\mu_{\left(N-1\right)}\times (N-1)+F_{N}}{N}\\ \begin{array}{cc} v_{N} & =\frac{v_{\left(N-1\right)}\times (N-1)+F_{N}^{2}}{N} \end{array}\\ \sigma_{N}^{2}=v_{N}-\mu_{N}^{2} \end{array}$$

In Equation (3), *N* is the number of collected data for time *t*, *μ_N_* is the mean of data *N*, *v_N_* is the mean of the square of data N and $\sigma_{N}^{2}$ is the distribution. If the number of calculated means and distributions is*j*, the proposed A-NB approach calculates the total mean value by combining *μ*_1_ to *μ_j_*, the total distribution value by the mean value of $\sigma_{1}^{2}$ to $\sigma_{j}^{2}$. In order to calculate the *p*′(*F_i_*|*C*) value, Equation (2) is transformed to Equation (4):

(4) $$P^{\prime }(F_{i}=v|C)=\frac{1}{\sqrt{2\pi \mu_{v}}}e^{-\frac{{\left(\nu -\mu_{m}\right)}^{2}}{2\mu_{v}}}$$

*μ_m_* is the mean value of*μ*_1_…*μ_j_*, *μ_v_* is also the mean value of the distribution $\sigma_{1}^{2}\cdots \sigma_{j}^{2}$. Hence the mean and distribution are calculated by data sample*F_i_*, and by using these values the posteriori probability *p*′(*F_i_* = *v*|*C*) are able to be calculated.

### 3.2. Lightweight Hierarchical Activity Recognition Framework (HARF)

Although the AR using multimodal sensors can increase recognizable activities and enable the recognition of various situations, it lowers the accuracy of the overall recognition result given that the classifier is required to consider more factors from the input data. In order to overcome this issue, HARF, which recognizes activities in a hierarchical approach, has been proposed. The approach includes the ability to not only recognize a simple activity, however, also to consider the spatial location of the users and their activity within a given context. The approach also considers that there may be different meanings associated with the user's location. The activities are categorized into three types, as presented in Table 1. There are 15 activities described in Table 1. Ten of them, for example, Walking, Sitting, Standing and Jogging are related to physical movement. Even though the physical action is the same in different locations, the pattern of movement or contextual information of the actions are totally different. For example, the moving pattern or Degree of Freedom (DoF) of walking at home and outdoors are obviously different. Also from a contextual point of view, we can say sitting at home as ‘taking a rest’, however, we can also assume the same physical action sitting at office as ‘working’, so four different physical activities are categorized according to the location where the action is recognized.

**Figure 4.**
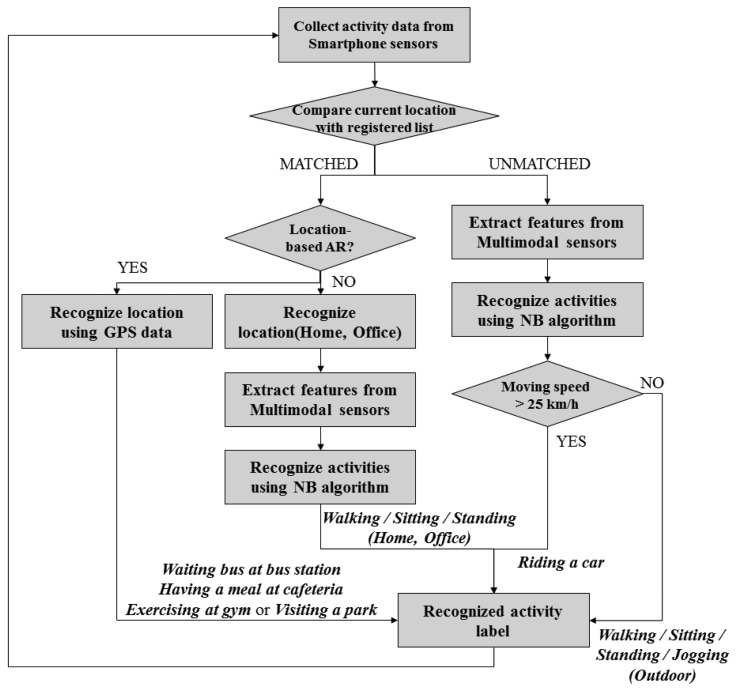
Hierarchical AR framework for enhancing recognition accuracy.

Figure 4 depicts the proposed HARF architecture for real-time AR processing based on the A-NB algorithm. If the A-NB is applied to AR, classification is performed firstly using location information and a heuristic approach is applied as described in Table 1. For recognizing the location of the user, we only used the GPS sensor embedded in a smartphone. In Type 1, location & multimodal sensor-based AR, the GPS is utilized to decide if the user is located indoors or outdoors. We determine that if the GPS signal is available and strong enough, the user is located in an outdoor area. We did not consider any other indoor locatization methdologies such as RSS using WLAN, Bluetooth or ZigBee *etc.* because we only need to differentiate whether the user is in Home/Office or Outdoors. In case of the Type 2, location-based AR, the GPS is a dominant sensor to recognize a current activity among four different location activity labels. In Type 2, GPS is the only option because specific coordinates are required to classify the location.

Once recognition is performed, the system recognizes the location first for differentiating indoors (Home and Office) and outdoors. If the user is at an unregistered location, the system tries to recognize the current activity among outdoor activities (Walking, Sitting, Standing, Jogging and Riding a Car) with a heuristic-based approach. Nevertheless, if the user is at a registered location, the system firstly looks up the location-based activity list. If the user is at home or the office, the system tries to recognize the activity using the proposed approach with multimodal sensor data.

As described in Figure 4, the HARF firstly compares the current location with the registered location list. If the current location is matched with one of the existing lists, the activity is categorized as indoor activities of Type 1 or Type 2 as described in Table 1. If not, the activity is recognized as ocurring in an outdoors area where it can then be one of four physical activities or riding a car. For recognizing a Type 3 activity, we used the moving speed information from GPS sensor data. Whatever physical activities are recognized using the proposed A-NB algorithm, if a moving speed of the user is over 25 km/h, it is compulsorily labeled as ‘riding a car’ activity. We named this intended labelling approach a Heuristic based activity recognition of our proposed HARF. By applying the heuristic approach, we can easily recognize the activity, riding a car, even when the activity can be misclassified if confused with several other physical activities such as sitting or standing. According to our experimental experiences, riding a car activity is somtimes recognized as Walking or even Jogging due to the road conditions without a heuristic methodology.

## 4. Implementation of the A-NB Based HARF

**Figure 5.**
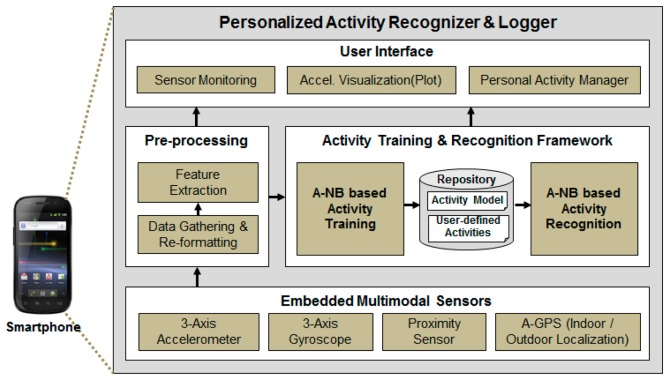
pARNL: Real-time activity recognition system using A-NB and HARF.

For evaluating the proposed algorithm in a smartphone environment, a real-time activity training and recognition system referred to as Personalized Activity Recognizer and Logger (pARNL) is proposed. Implementation using the pARNL enables personalized activity recognition and life-logging and users can add or monitor their own activities or contexts. pARNL is organized into four sub-modules as described in Figure 5.

The component, *Embedded Multimodal Sensors*, consists of four sensors (3-axis accelerometer, 3-axis gyroscope, proximity sensor and GPS) embedded in the smartphone for recognizing activities. The *Pre-processing* component collects sensing data from multi-modal sensors periodically (50 Hz) and unifies the data format for efficient data processing sharing. It also extracts features from collected sensor data for activity modeling and recognition. The *Activity Training & Recognition Framework* is composed of the proposed A-NB algorithm-based activity training and recognition modules. Activity models and user-defined activities are stored at the Repository. Lastly, the *User Interface* permits the users to monitor the collection of data from multimodal sensors and visualize 3-axis accelerometer data. It also provides an interface for users to add or train their own activities.

For an implementation of the pARNL system, a smartphone which has four available sensor modules: 3-axis accelerometer, 3-axis gyroscope, proximity sensor and GPS, is required. We used a Samsung Galaxy S3 cellphone for our evaluation. The information below is the detailed specifications of the device related to our proposed system:

- Display: Super AMOLED capacitive touch screen
- Internal ram size: 1 GB
- Network connectivity: Not required
- OS: Android V4.3(Jelly Bean) but successfully tested on the lower version 2.3.3 (API level 10)
- CPU and GPU : Quad-core 1.4 GHz Cortex-A9 / Mali-400MP4
- GPS: Assisted GPS, GLONASS

## 5. Performance Evaluation

**Figure 6.**
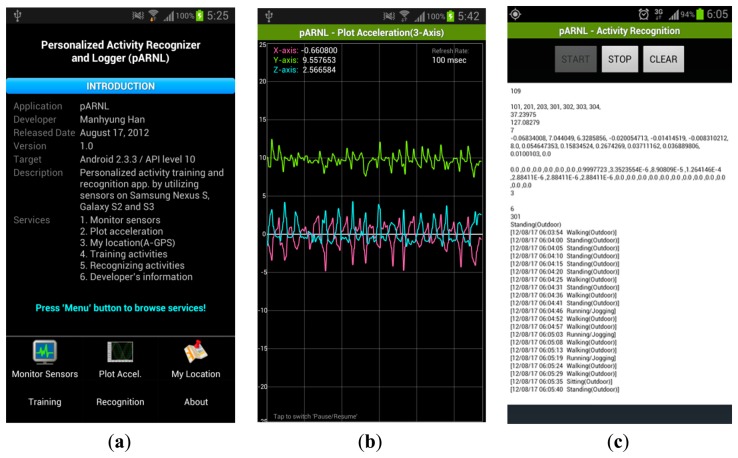
Smartphone application which implements real-time activity recognition framework. (**a**) Initial state of application. Sensor monitoring, accelerometer visualization, UI for activity training & recognition; (**b**) Visualizing 3-axis accelerometer values of walking activity; (**c**) Screenshot of activity recognition results.

For the verification of the A-NB algorithms and the HARF framework, a real-time AR system has been implemented in the form of a smartphone application using the Android OS. The application which uses the Android OS can be installed easily on a smartphone or mobile device. In addition, a variety of smartphone UI (touch screen, keyboard, sound, *etc.*) enables to the users to add or model their own activities by themselves. The smartphone application was developed based on Android 2.3.3 (API level 10), as shown in Figure 6, on the Samsung Nexus S model.

Table 2 presents the results of the AR using the smartphone application. The experiments were conducted on 15 activities including four location-based activities (waiting for bus at bus stop, having a meal at a cafeteria, exercising at gym, visiting a park). Nevertheless, the results of recognizing these four location-based activities are not presented. There was only one misrecognized case out of 200 testing samples. Nevertheless, if the GPS on the smartphone is guaranteed to work well, location-based activities are recognized well in the proposed system. Therefore, the experimental results in Table 2 present the accuracy of 11 activities without location-based activities included.

The recognition results of the 15 activities provided an accuracy of 92.96% and the result of 11 activities, without including the activities based on only location, was 90.4%. There are several cases which show different accuracies for the same activities. This indicates that the activity can be recognized differently depending on where the activities took place. For example, walking activities in the home or outdoors are seldom recognized as a standing activity given that the user is frequently stopped to change direction. The recognition accuracy of both sitting and standing activities are relatively higher than the others due to their static characteristics.

**Figure 7.**
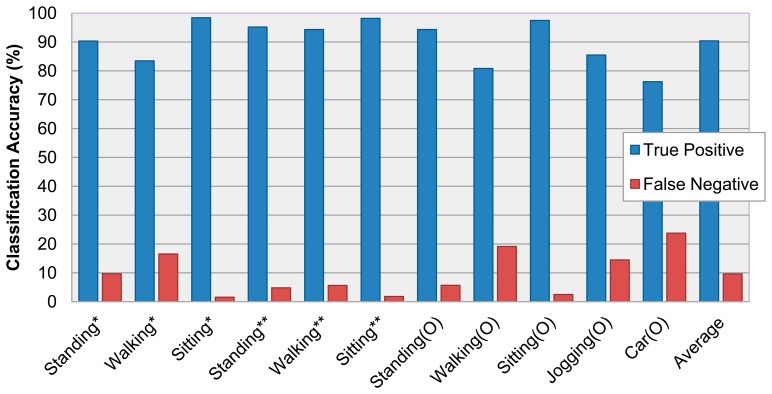
Activity recognition accuracy graph of 11 activities (*: Home, **: Office, O: Outdoors).

In the case of jogging and riding in a car, there are some misrecognition results given that a jogging activity is similar to walking and a car is frequently stopped or driven slowly. Figure 7 depicts True Positive and False Negative of the 11 activities based on Table 2.

**Figure 8.**
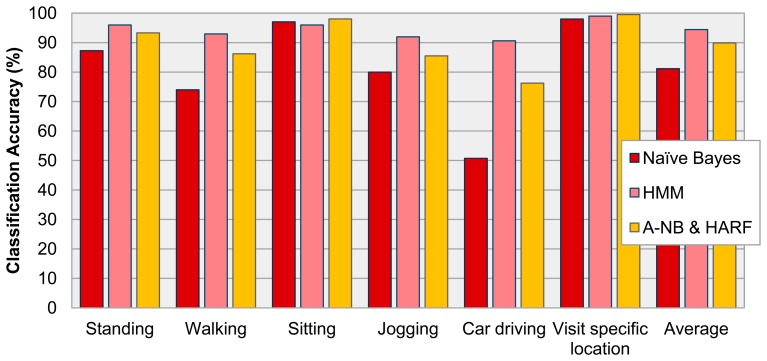
Accuracy comparison of Naïve Bayes, HMM and HARF for 15 activities.

A performance comparison of HARF, HMM and Naïve Bayes algorithm is presented in Figure 8. The Hidden Markov Model is commonly used in offline processing and it shows more accurate performance, however our proposed algorithm combination, A-NB and HARF, is running on a resource limited mobile environment for the purposes of real-time processing. The average accuracy of recognizing 15 activities (including four location-based activities) using Naïve Bayes and HMM was 81.17% and 94.42% respectively. On the other hand, the proposed HARF provided 89.88% accuracy. This means that the proposed algorithm and framework enhanced the Naïve Bayes and can replace the heavier but more accurate approach—HMM based methodology.

Among the average accuracies of three different approaches, HMM-based AR showed the best performance, however, the proposed HARF showed better performance on ‘sitting’ and ‘visit specific location’ activities than HMM. Also in the case of recognizing Car Driving, HARF and HMM showed 76% and 90% accuracy, however, the Naïve Bayes showed much lower accuracy of around 50%. The result means that a complex activity such as ‘car driving’ is recognized well using the powerful and heavy algorithm, HMM, however, the proposed HARF recognizes simple activities such as ‘sitting’ and ‘visit specific location’ and others better.

## 6. Conclusions and Future Work

In this paper, we have proposed a personalized activity modeling and real-time AR framework for understanding a human's intention or requirements based on multimodal sensors in a smartphone. A Hierarchical Activity Recognition Frame-work for modeling and recognizing user activities in a resource restricted smartphone was proposed. For compensating the memory overflow errors which may occur within the constrained resources of the smartphone when considering data from multimodal sensors, we have proposed a hierarchical activity modeling and recognition approach. Results following testing demonstrate that the proposed system can recognize 15 activities with an accuracy of 92.96%. This is 10.73% higher than using a conventional Naïve Bayes approach.

The proposed HARF exhibited relatively high accuracy when recognizing simple or fixed activity patterns (jogging, standing). In the case of activities such as riding a car or walking, which may have various patterns, it showed lower accuracy. In order to compensate for these problems, other studies using not only a smartphone, however, also external sensor devices or utilizing environmental sound are required. A compound approach utilizing multimodal sensor data and external sensor data is expected to enhance the accuracy of activity modeling and recognition.

Our current implementation of the proposed HARF uses four sensor modules all the time when the application is running. This means each sensor module is generating raw sensor data continuously and the system is being required to process everything. One of our future planned works is to optimize a proposed hierarchical framework. For example, we first enable only the GPS sensor for classifying the current activity based on location. If other sensor data are required to classify detailed activities; we turn on the required sensors one by one. By applying the optimization approach, battery consumption may be naturally reduced.

## Figures and Tables

**Table 1.** Activity categorization for hierarchical AR.

**Type**	**Area**	**Activity**	**Sensors**
**Location & multimodal sensor based activity recognition**	Home	Walking	Accelerometer, Gyroscope, Proximity and GPS
		Sitting	
		Standing	

	Office	Walking	
		Sitting	
		Standing	

	Outdoor	Walking	
		Sitting	
		Standing	
		Jogging	

**Location based activity recognition**	Outdoor	Waiting for bus at bus stop	GPS
		Having a meal at cafeteria	
		Exercising at gym	
		Visiting a park	

**Heuristic based activity recognition**	Outdoor	Riding a car	Accelerometer, Gyroscope, Proximity, GPS and Heuristic

**Table 2.** Activity recognition accuracy of 11 activities for validating the proposed HARF.

**Location**		**Home**			**Office**			**Outdoor**				

		**Standing**	**Walking**	**Sitting**	**Standing**	**Walking**	**Sitting**	**Standing**	**Walking**	**Sitting**	**Jogging**	**Car**
**Home**	Standing	**90.32**	-	9.68	-	-	-	-	-	-	-	-
	Walking	10.43	**83.47**	6.1	-	-	-	-	-	-	-	-
	Sitting	2.56	-	**98.44**	-	-	-	-	-	-	-	-
**Office**	Standing	-	-	-	**95.2**	-	4.8	-	-	-	-	-
	Walking	-	-	-	4.84	**94.35**	0.81	-	-	-	-	-
	Sitting	-	-	-	1.2	0.61	**98.19**	-	-	-	-	-
**Outdoor**	Standing	-	-	-	-	-	-	**94.34**	-	5.66	-	-
	Walking	-	-	-	-	-	-	12.77	**80.85**	6.38	-	-
	Sitting	-	-	-	-	-	-	2.5	-	**97.5**	-	-
	Jogging	-	-	-	-	-	-	2.17	10.86	1.47	**85.5**	-
	Car	-	-	-	-	-	-	16.25	6.25	1.25	-	**76.25**
